# Management of Yellow Phosphorus-Induced Acute Liver Failure: A Case Report and Review of Literature

**DOI:** 10.7759/cureus.54223

**Published:** 2024-02-15

**Authors:** Rohini R, Manender Routray

**Affiliations:** 1 Medicine, All India Institute of Medical Sciences, Raipur, IND; 2 General Medicine, All India Institute of Medical Sciences, Raipur, IND

**Keywords:** n-acetyl cysteine, yellow phosphorus poisoning, plasmapheresis, liver transplantation, acute liver failure

## Abstract

Three percent (3%) of yellow phosphorus is the active component of the rodenticide Ratol^®^. It is a potent hepatotoxin that leads to acute liver failure (ALF) with high mortality. There is no antidote available; the only definitive management is liver transplantation. Therapeutic plasma exchange, or plasmapheresis, appears to help these patients by removing the toxin, its metabolite, or the inflammatory mediators released in the body in response to the toxin. Here, we report a case of a 19-year-old male with an alleged history of Ratol^®^ ingestion and ALF with acute kidney injury. He had a complete reversal of his condition with timely intervention in the form of plasmapheresis.

## Introduction

Yellow phosphorus is a compound used in match industries, fireworks industries, and rodenticide products. Ingestion of yellow phosphorus by humans leads to toxicity [1]. It is a non-metallic protoplasmic poison. Once consumed, it gets absorbed rapidly from the gastrointestinal tract and is metabolized mainly by the liver [2]. The minimal fatal dose is 8 mg. The dose leading to death in 50% of individuals (LD50) is around 1 mg/kg [3]. In India, consumption of yellow phosphorus-containing rodenticide for suicidal ideas is relatively common, with a fatality rate of more than 50%, especially among those who present with complications [4]. In our center alone, rodenticide poisoning accounted for 11.7% of all poisoning cases [5]. There are no approved antidotes for yellow phosphorus-induced acute liver failure (ALF), unlike in acetaminophen poisoning, where high-dose N-acetyl cysteine (NAC) is prescribed as the antidote. However, even in acetaminophen poisoning, the therapy with NAC needs to be started early, defined as less than 8-10 hours after ingestion of acetaminophen [6]. Here, we present the case of a 19-year-old male patient who ingested Ratol® paste, containing yellow phosphorus. He presented to us four days after ingestion with ALF. We report this case to emphasize the need for the rapid institution of plasma exchange (PLEX) as a life-saving measure.

## Case presentation

A 19-year-old male presented to the emergency department after four days of ingestion of about 35 grams of Ratol® paste. He did not mix the paste with any other substance for consumption. Prior to presentation to our center, he had not consulted any medical center, as he was asymptomatic. He developed nausea, vomiting, decreased appetite, and loose stools on the fourth day after ingestion. At presentation, his pulse was 70/min, his blood pressure was high at 156/78 mm Hg, and his respiratory rate was 18/min. He was in grade 2 hepatic encephalopathy with a GCS of E3V4M5. On examination, an icterus was present. He did not have any obvious bleeding from any site. His abdomen, cardiovascular, and respiratory system exams were within normal limits. His initial investigations showed severely deranged liver function tests (LFTs) with coagulopathy. He also had elevated serum creatinine levels, indicating acute kidney injury (AKI). Table 1 shows his initial investigation reports on the day he presented them to us, that is, four days after the ingestion of the toxin.

**Table 1:** Initial investigations on presentation

Laboratory parameters	Patient’s value	Normal range
Hemoglobin	13 g/dL	12-16 g/dL
Total leukocyte count	5,150/µL	4,000-10,000/µL
Platelet count	95,000/µL	150,000-450,000/µL
Urea	73 mg/dL	15-40 mg/dL
Creatinine	2.8mg/dL	0.9-1.3 mg/dL
Total bilirubin	17.2 mg/dL	0.3-1.3 mg/dL
Direct bilirubin	9.3 mg/dL	0.1-0.4 mg/dL
Aspartate transaminase	112 U/L	12-38 U/L
Alanine transaminase	114 U/L	7-41 U/L
Activated partial thromboplastin time	47.8 s	23.2-32.4 s
International normalized ratio	4.2	0.8-1.4

**Figure 1:**
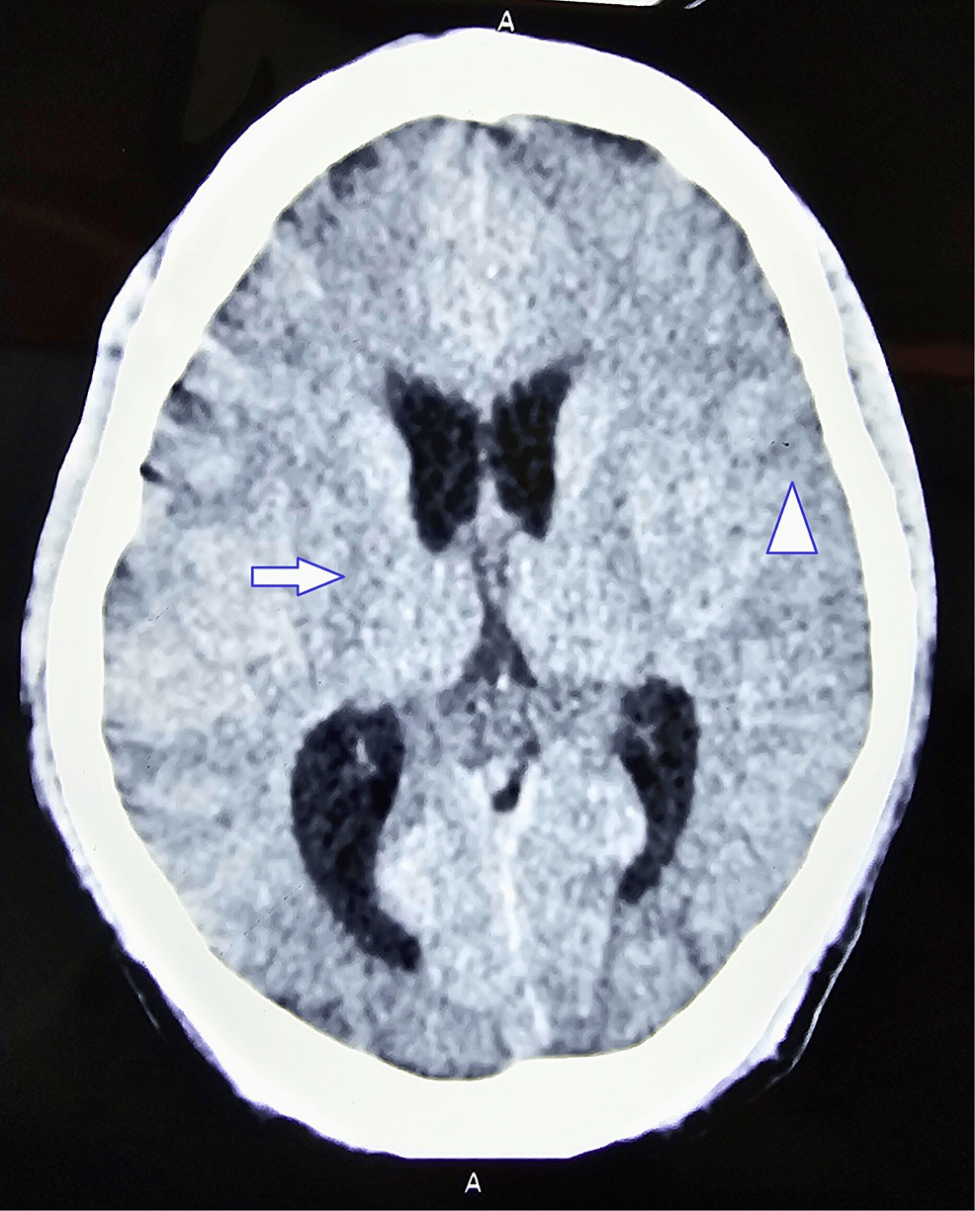
Non-contrast computed tomography head showing cerebral edema Arrow showing poor grey-white differentiation. Arrowhead showing obliteration of sulci. Both features of cerebral edema.

Non-contrast computed tomography of the head revealed gross cerebral edema (Figure 1). With the above clinical picture, a diagnosis of fulminant hepatic failure with AKI secondary to yellow phosphorus toxicity was made. Standard of care treatment was started in the form of intravenous fluids, vitamin K, 3% normal saline for cerebral edema and lactulose syrup, and rifaximin for hepatic encephalopathy. Though NAC has no clear indication after the onset of ALF, the patient was given the benefit of the doubt and started on injection NAC 150 mg/kg in 5% dextrose as a bolus, followed by 50 mg/kg over four hours, followed by 100 mg/kg as a continuous infusion for 16 hours, as per protocol for acetaminophen-induced ALF.

However, he did not show any improvement; rather, the patient was deteriorating rapidly. A decision was made to start him on plasmapheresis. He underwent five cycles of plasmapheresis. It was done using both fresh frozen plasma (FFP) and human albumin. Low-volume plasmapheresis was done using a plasma volume of 40 ml/kg, and 100 ml/20 g of human albumin was given for every 500 ml of plasma in case of non-availability of FFP. His clinical status started showing improvement after two cycles of plasmapheresis. Repeat serum investigations also followed, significantly improving LFT, coagulopathy, and RFT parameters. Table 2 shows the trend of his lab investigations over the past two weeks.

**Table 2:** Trend of investigations during the hospital stay PLEX: plasma exchange

Laboratory parameters	Post two cycles of PLEX (day 7)	Post four cycles of PLEX (day 12)	Post five cycles of PLEX (day 15)	Normal values
Hemoglobin	14 g/dL	10.9 g/dL	9.9 g/dL	12-16 g/dL
Platelets	97,000/µL	104,000/µL	231,000/µL	150,000-450,000/µL
Creatinine	2.01 mg/dL	0.69 mg/dL	0.62 mg/dL	0.9-1.3 mg/dL
International normalized ratio	1.8	1.1	1.1	0.8-1.4
Aspartate transaminase	114 U/L	110 U/L	85 U/L	12-38 U/L
Alanine transaminase	112 U/L	99 U/L	74 U/L	7-41 U/L
Total bilirubin	13.6 mg/dL	7.63 mg/dL	4.78 mg/dL	0.3-1.3 mg/dL
Direct bilirubin	7.5 mg/dL	3.98 mg/dL	2.34 mg/dL	0.1-0.4 mg/dL

The patient maintained his improved status with no evidence of coagulopathy, hepatic encephalopathy, or AKI, and was discharged. He was reviewed in follow-up after a month and maintained his status. He is currently on a monthly follow-up and is doing well.

## Discussion

Phosphorus is available in three forms, namely red, white, and yellow. Yellow phosphorus is used predominantly in match industries, firecrackers, and as rodenticides [1]. Exposure is usually either intentional with suicidal ideation or accidental.

Yellow phosphorous is a protoplasmic toxin. It causes deterioration in the function of the liver, kidney, gastrointestinal, and cardiovascular systems. It is absorbed through the gastrointestinal tract and respiratory mucosa. Serum phosphorous levels rise quickly after consumption of yellow phosphorus. The proposed mechanism of toxicity states that it affects ribosomal function, thus leading to defective protein synthesis. It impairs glucose homeostasis and lipoprotein and triglyceride metabolism, which in turn leads to fatty degeneration of the liver and kidney [7,8].

The clinical presentation after ingestion of yellow phosphorus can be divided into three stages [8]. The first stage presents within the initial 24 hours as either asymptomatic or with mild gastric discomfort. The second stage presents within 24-72 hours after ingestion, and there is resolution of symptoms. The third stage presents itself after 72 hours of ingestion. The patient usually presents with ALF and AKI in this stage, which is often fatal. Our patient presented in the third stage, four days after ingestion.

The initial treatment includes decontamination and supportive therapy. These patients need to be monitored in an intensive care unit. Liver and renal function tests should be done daily [9]. NAC is well documented for paracetamol poisoning and has also been studied for other causes of ALF [10]. There are case reports and case series of rodenticide poisoning that have shown that NAC may be useful in cases of rodenticide-induced ALF, but further evidence is needed to show the clear advantage of NAC in such cases. Table 3 shows various studies that have studied the effect of NAC on rodenticide poisoning.

**Table 3:** NAC in yellow phosphorus poisoning NAC: N-acetyl cysteine

Si. No.	Study	Site	Type of article	Sample size	Conclusion
1	Mark K, Hyder S, Rashid M, Chandran VP, Seshadri S, Seshadri S, Nair S, Thunga G. Survival Benefits of N-Acetylcysteine in Rodenticide Poisoning: Retrospective Evidence from an Indian Tertiary Care Setting. Curr Rev Clin Exp Pharmacol. 2021;16(2):201-208 [11]	India	Retrospective	229	NAC was shown to have significant survival benefits with favorable safety profile
2	Fernandez OU, Canizares LL. Acute hepatotoxicity from ingestion of yellow phosphorus-containing fireworks. J Clin Gastroenterol. 1995 Sep;21(2):139-42. [12]	Philippines	Retrospective	15	NAC did not confer a significant survival benefit
3	Dawra S, Kumar A, Kumar D, Ari B, Srivastava S, Manrai M. Rodenticide-induced acute liver failure - Uncommon presentation of commonly available poison. Trop Doct. 2021 Oct;51(4):561-565 [13]	India	Case series	3	NAC probably conferred a survival benefit

For most patients with ALF secondary to yellow phosphorus poisoning, a liver transplant is the definitive treatment [13]. The American College for Apheresis recommends high-volume PLEX, or plasmapheresis, as the first-choice therapy for ALF of any etiology [14]. Various studies have shown the benefit of PLEX in ALF secondary to different etiologies, including paracetamol poisoning [15], viral hepatitis [16], Wilson’s disease [17], and rodenticide poisoning among children [18]. However, no such study has been done among adults with yellow phosphorus poisoning.

Plasmapheresis, or PLEX, acts similarly to renal replacement therapy, as in, taking over the main functions of the liver. It detoxifies the circulation by removing the proinflammatory cytokines, which originally led to multi-organ failure. The fluid used for the replacement of the patient's plasma restores the coagulation factors, albumin, and immunoglobulins, thus taking over the synthetic function of the liver. This allows the liver to rest and gives it time to regenerate. The complete process improves the microenvironment of the liver, which further accelerates regeneration and helps in its functional recovery [19]. It is usually used as a bridge to liver transplantation [4]. An Indian study done in Vellore, India, recruited 18 children with rodenticide poisoning. It showed that children who underwent PLEX had a better three-month survival rate than those who did not receive PLEX [18]. The benefit of PLEX can be monitored by serial LFTs, as can the clinical status of patients. The delta model for end-stage liver disease may be used as a prognostic indicator of outcome [19].

Our patient presented with ALF and AKI. Though we initiated him on an NAC infusion, he was rapidly deteriorating, and hence, a decision was taken to initiate PLEX. He showed rapid improvement clinically as well as in his lab parameters, notably serum bilirubin, coagulopathy, and creatinine, which were sustained even after stopping the cycles of PLEX. Our patient benefitted from five cycles of PLEX and was discharged.

## Conclusions

PLEX has been approved by the American College for Apheresis as a first-line therapy in ALF. However, its status as a treatment modality in ALF as per the hepatology and gastroenterology societies remains undefined. As per our case study, there was a drastic improvement in the clinical as well as lab parameters of the patient after the initiation of PLEX. PLEX shows promise as an effective non-liver transplant treatment in patients with yellow phosphorus-induced hepatotoxicity. A prompt decision to resort to PLEX, especially in resource-limited settings where liver transplants are not readily available, can help save several lives.
